# Orthognathic Surgery Treatment Need in a Turkish Adult Population: A Retrospective Study

**DOI:** 10.3390/ijerph16111881

**Published:** 2019-05-28

**Authors:** Hatice Kübra Olkun, Ali Borzabadi-Farahani, Sina Uçkan

**Affiliations:** 1 Department of Orthodontics, School of Dentistry, İstanbul Okan University, İstanbul 34959, Turkey; kubra.olkun@okan.edu.tr; 2 Orthodontics, Department of Clinical Sciences and Translational Medicine, University of Rome Tor Vergata, 00183 Rome, Italy; 3 Finchley Orthodontics, North Finchley, London N12 9EN, UK; 4 Department of Oral and Maxillofacial Surgery, School of Dentistry, İstanbul Medipol University, İstanbul 34214, Turkey; isuckan@medipol.edu.tr

**Keywords:** craniofacial anomalies, orthognathic surgery, IOFTN

## Abstract

*Objectives:* Limited information exists on orthognathic procedures and respective dentofacial deformities in Turkey. This retrospective study assessed the orthognathic surgery procedures in two universities, using the Index of Orthognathic Functional Treatment Need (IOFTN), and compared the IOFTN grades according to gender as well as sagittal and vertical skeletal relationships. *Material and Methods:* Records of 200 consecutive patients (120 females, 80 males, mean age = 23.4 (SD: 5.4) years) who received orthognathic treatment (2014–2018) were analyzed. Sagittal (ANB angle) and vertical skeletal type (GoGnSN angle), osteotomies, and IOFTN scores were recorded. *Results:* Class III, II, and I malocclusions formed 69%, 17.5%, and 13.5% of the samples, respectively. Class III skeletal relationships (69%) and high-angle cases (64%) were the most prevalent (*p* < 0.05). IOFTN scores were unevenly distributed among genders (*p* < 0.05) and the prevalent scores were 5.3 (40.5%), 4.3 (15.5%), 5.4 (13%), and 5.2 (7.5%), with 94% scoring 4 or 5 (great and very great functional need). Bimaxillary osteotomies were the most prevalent (55%), followed by LeFort I (32%), and 26% had genioplasty. *Conclusion:* IOFTN is a reliable tool to identify patients in need of orthognathic surgery. Class III malocclusions and Class III sagittal skeletal relationships were more common in this sample. Comparatively, a higher number of patients had genioplasty as a part of their treatment.

## 1. Introduction

Dentofacial deformity is defined as significant deviations from normal proportions of the maxillo mandibular complex and is usually accompanied by a malocclusion [1,2,3]. This can adversely affect the head and neck functions as they relate to breathing, swallowing, speech articulation, and chewing [2]. Literature suggests that nearly 5% of the population is affected by dentofacial deformities and need orthognathic surgery as a part of their definitive treatment [1,2,3].

Orthognathic surgery, usually combined with orthodontic treatment, is a common treatment alternative in the management of dentofacial deformities [1]. Athanasiou et al. [4] reported that the major concerns for orthognathic surgery were surgical risks (36%), change of appearance (27.6%), and finances (15.8%). In recent years, development of new surgical techniques as well as changes in the dentofacial aesthetic perception of the physicians and patients, and approval for health insurance coverage for surgery and hospitalization collectively increased the demand for orthognathic surgery [5,6].

The need for orthognathic surgery can vary according to the perception of patients and physicians and may depend on self-confidence, appearance, and oral function [7,8]. Publicly funded institutions need a method to determine who should be primarily covered for surgery. Objective evaluation of dentofacial deformities is important in determining the severity and prevalence of deformity in the population, as well as determining the allocation of the national budget towards treatment, especially for high priority treatments. This may involve regionalization of patients to high-volume experiences surgeons to decrease the incurred costs [9]. For this reason, such treatments require measures or indices that can accurately and objectively assess the need for treatment. In order to make the correct assessment, many indices have been developed in the field of orthodontic treatment need; however, until 2014 there had not been an index for the assessment of orthognathic surgery need [10,11].

Reports on the types of orthognathic surgeries in Turkey are non-existent, and insight will help resource planning in the future. In Turkey, the Social Security Institution (SSI) provides some funding for orthognathic surgeries. For this purpose, no index has been used to determine the need for orthognathic surgery and SSI payment. There are certain fees that the government determines for different types of orthognathic surgery if there is a functional need (sleep apnea, chewing difficulty) for surgery. The government does not accept operations for aesthetic purposes only. For this, the opinion of the oral and maxillofacial surgeon is usually taken into account. In 2014, Ireland and colleagues [11] developed the Index of Orthognathic Functional Need (IOFTN). This index has demonstrated good validity and very good inter-operator agreement in different ethnic populations. It is known that geographic location may affect orthodontists’ determination of treatment need [12,13]. Therefore, the aims of this retrospective study were to provide insight on the profile of orthognathic surgery procedures in a Turkish adult sample, categorize these patients using the IOFTN and assess its applicability to prioritize the funding for these patients, and compare the IOFTN grades according to gender as well as sagittal and vertical skeletal relationships.

## 2. Methods

The Ethics Research Committee of the İstanbul Medipol University approved this retrospective study (10840098-604.01.01-E.50019; 12/11/2018). Records of 200 consecutive patients (120 females, 80 males; 18–50 years of age, mean (SD) = 23.4 (5.4)) who had undergone orthodontics and orthognathic surgery in the years 2014–2018 at the Department of Oral and Maxillofacial Surgery at İstanbul Medipol University and the Department of Orthodontics of İstanbul Okan University were analyzed. The patients were evaluated by a single examiner (first author) using their study models, photographs, and radiographs. The IOFTN score was recorded and evaluated by the second author (ABF) and used to determine the orthognathic treatment need of each patient. To assess intra-examiner reliability, the records of 20 patients were randomly selected and re-examined 2 weeks after their initial examination. The kappa value was 0.99 for intra-rater reliability. Patients were classified according to IOFTN scores, sagittal skeletal relationship, vertical skeletal relationship, type of osteotomy, and gender. The overjet and overbite were also recorded.

### 2.1. Index of Orthognathic Functional Need (IOFTN)

IOFTN is valid for patients who have completed facial growth prior to surgery (18 years of age or older). The index consists of five categories (Very Great Need, Great Need, Moderate Need, Mild Need, and No Need) and each category has subgroups. Assessment begins at the fifth category and ends at the first category. The full detail of the IOFTN scoring system is available on the open access journal [11].

### 2.2. Sagittal and Vertical Skeletal Relationship

In order to determine the sagittal skeletal relationship, the patients were divided into three groups according to their ANB angles as Class I (1 ≤ ANB ≤ 4), Class II (ANB > 4), and Class III (ANB < 1). For assessing the vertical skeletal relationship, the patients were divided into three groups according to their GoGnSN angles as normal (28 ≤ GoGnSN ≤ 36), high-angle (GoGnSN > 36), and low angle (GoGnSN < 28).

### 2.3. Osteotomy Type

The types of osteotomy applied to patients were bimaxillary surgery, LeFort I, bilateral sagittal split osteotomy (BSSO), and genioplasty.

### 2.4. Statistical Analysis

The SPSS statistical software (SPSS version 18.0; SPSS Inc., Chicago, IL, USA) was used to perform statistical analysis. Chi-square tests were used to assess the statistical significant gender differences as well as to detect IOFTN treatment category differences among various sample subcategories such as malocclusions, and patients with different sagittal and vertical relationships. Level of significance was set at *p* < 0.05.

## 3. Results

Class III was the most prevalent type of malocclusion (69%) observed in this study, followed by Class II (17.5%) and Class I (13.5%). In terms of sagittal and vertical skeletal relationships, Class III skeletal (69%) and high-angle cases (64%) were the most prevalent types (*p* < 0.05, Table 1). The most prevalent IOFTN score was 5.3 (40.5%), followed by 4.3 (15.5%), 5.4 (13%), and 5.2 (7.5%). IOTFN scores were not equally distributed between genders (*p* < 0.05) (Table 2). The female sample had more patients that scored 3.10, 4.2, and 5.2. Overall 94% of orthognathic surgery patients were classified as having great and very great functional need.

The gender distribution of sagittal skeletal relationships was different (*p* = 0.008), with females having more Class II skeletal pattern (sagittal); however, this was not the case when we looked at vertical skeletal relationships (*p* = 0.174) (Table 3). We saw gender differences when IOFTN scores were re-grouped to grades 5, 4, and ≤3 (*p* = 0.038, Table 4); similarly, IOFTN scores were not equally distributed in patients with various sagittal skeletal relationships (*p* = 0.001), but this was not the case when we looked at vertical skeletal relationships (*p* = 0.576) (Table 5).

Bimaxillary was the most prevalent osteotomy type (55%), followed by LeFort I (32%). Genioplasty was used in 26% of patients (Table 6). Five patients had distraction osteogenesis (maxillary expansion) before receiving orthognathic surgery.

## 4. Discussion

IOFTN is a newly developed index, and a limited number of studies have been published on IOFTN so far [14,15,16,17,18,19]. Harrington et al. [19] conducted a retrospective study using the data of Northampton General Hospital in the UK and reported that the most prevalent IOFTN score was 5.2 (increased overjet > 9 mm), followed by 5.3 (reverse overjet ≥ 3 mm), and 4.2 (increased overjet ≥ 6 mm and ≤ 9 mm). Borzabadi-Farahani et al. [17] also conducted a study using the data of a teaching university hospital in Iran and reported that the most prevalent IOFTN score was 5.3, followed by 4.2 and 4.3 (reverse overjet ≥ 0 mm and < 3 mm with functional difficulties). In the present study, the most prevalent IOFTN score was 5.3 (reverse overjet ≥ 3 mm), followed by 4.3 (reverse overjet ≥ 0 mm and < 3 mm with functional difficulties), and 5.4 (open bite ≥ 4 mm). Referring clinicians may use the IOFTN to see if patients are suitable for orthognathic treatment [16]. The acronym OOSGA (Overjet, Overbite, Scissors bite, Gingival exposure, and Asymmetry) has been suggested to improve the efficiency of scoring patients [15]; this is similar to the IOFTN’s hierarchy allocation system (MOCDO, missing teeth, overjet, crossbite, displacement of contact point, overbite) [10] and would cover the majority of the subcategories within IOFTN, thus helping to identify the single worst feature of the patient’s malocclusion.

Maxillo-mandibular advancement is used in the management of obstructive sleep apnea to improve form and function of the upper airway [20]. Proffit et al. [5] reported that Class I patients in Alabama increased from 5% to 26% in years, and most of them were treated via rotate occlusal plane or maxillo-mandibular advancement to control sleep apnea. Different from previous indices, the IOFTN also considers the presence of sleep apnea (score 5.6). Similar to other studies, there were no patients who underwent orthognathic surgery merely because of sleep apnea [17,18,19]. In the case of sleep apnea, genioplasty is sometimes applied for functional purposes. Castro et al. [21] found a high frequency of chin surgery (76.86%) either isolated or in combination with maxillary or mandibular surgery. In this study, the prevalence of isolated genioplasty was 1%, and the prevalence of genioplasty in combination with other osteotomies was 26%, which is higher than the previously reported studies [19,22,23].

The prevalence rates of the different types of dentofacial deformities vary among nationalities. Lee et al. [24] compared the profiles of dentofacial deformity in Chinese and Caucasian patients and reported that Class III skeletal relationship was the most prevalent for both groups. In the present study, Class III and high-angle skeletal types were the most prevalent dentofacial deformities. Ruslin et al. [25] reported that the most common dentofacial deformity in Southeast Asian patients is mandibular prognathism with open bite. According to the present study, 38.5% of the patients had Class III with high-angle skeletal relationships.

Similar to other occlusal indices, there are some limitations using IOFTN. It does not assess the sagittal, vertical, and transverse skeletal relationships, which can be compensated by the teeth [14,17,26,27]. Another limitation is that there is no score in the IOFTN that only represents the chin deformity. In this study, two of the patients underwent only genioplasty due to anteroposterior chin deficiency and were scored as 1.14. A scoring that identifies this deformity (chin deficiency) could be added to the index. The retrospective nature of the presented data, as well as the exclusion of subjects with incomplete data, which resulted in a relatively small sample (200) and the possible existence of bias, is another limitation of the study. Future studies can use prospective analysis to assess if IOFTN identifies all patients in need of orthognathic surgery.

Similar to the other studies, a high percentage (94%) of this sample were scored as having great and very great functional needs [16,17,18,19,28], and most of the patients with Class III skeletal relationships were classified as grade 5 [16,17,18,19]. This study also revealed that most of the subjects with high-angle skeletal relationships (60%) were categorized as IOFTN grade 5 or 4.

In order to determine the orthognathic treatment need, the quality of life should be considered, as Ireland et al. suggested [11]. Patients with complex treatment or severe malocclusion may report various oral health impacts that affect their well-being in many ways. Previous research findings indicate that the perception of malocclusion varies across specialists and patients; additionally, the severity of dentofacial deformity does not always reflect oral health-related quality of life (OHRQOL) [29,30]. We know that OHRQOL tends to improve after orthognathic surgery in patients with dentofacial deformities [31]. The use of the OHRQOL measure as part of the diagnostic procedure may provide more information for prioritizing treatment in order to maximize patient satisfaction. As previously suggested, further investigations are required to clarify functional difficulties that are applicable to the IOFTN, and to find out the most appropriate self-reported OHRQOL measure(s) to complement the use of the IOFTN [32].

Orthognathic surgery in evolving and there are many reports on the use of distraction osteogenesis as a part of treatment, particularly when the necessary surgical movements are beyond the limits of conventional orthognthic surgery [33,34,35,36]. We did not identify cases that only underwent distraction osteogenesis without further orthognathic surgery. This was mainly to correct the maxillary transverse deficiency prior to orthognathic surgery. Reviewing previous studies it is not clear if they included cases with distraction osteogenesis as a part of dentofacial deformity correction [14,18,32].

In a nationwide study in the USA [23], among orthognathic surgery patients, 19.6% underwent concurrent ancillary procedures (genioplasty, rhinoplasty, or septoplasty), and 37.6% underwent bimaxillary surgery. In the present sample, concurrent genioplasty was used in much higher percentages of patients (26%), but 55% underwent bimaxillary surgery, which is higher than the figures reported in the USA [23]. The high incidence of bimaxillary surgery may reflect the high number of individuals with greater severity of dentofacial deformities that seek treatment. Previous studies in the UK also reported high incidence of bimaxillary surgery such as O’Brien et al., (66%) [37], Harrington et al., (61.5%) [19], and Cartwright et al., (63.1%) [14].

## 5. Conclusions

Within the limitations of this study, it was observed that IOFTN identified 94% of orthognathic surgery patients as having great and very great functional needs. The results indicated that IOFTN is a reliable tool to identify patients in need of orthognathic surgery. Class III malocclusions and Class III sagittal skeletal relationships were more common in this sample. A higher number of patients were identified that had genioplasty as a part of their treatment.

## Figures and Tables

**Table 1:** Distribution of vertical and sagittal skeletal relationships among study patients, *N* (%).

Sagittal Skeletal Relationship	Vertical Skeletal Relationship *			Total
	High-Angle	Low-Angle	Normal	
Class I	18 (14.1)	4 (8)	2 (9.1)	24 (12)
Class II	33 (25.8)	4 (8)	1 (4.5)	38 (19)
Class III	77 (60.2)	42 (84)	19 (86.4)	138 (69)
Total	128 (64)	50 (25)	22 (11)	200

* Assessed based on GoGnSN angles: normal (28 ≤ GoGnSN ≤ 36), high-angle (GoGnSN > 36), and low angle (GoGnSN < 28).

**Table 2:** Gender distribution of IOFTN subscores.

IOFTN Scores	Gender		*N* (%)
	Male	Female	
1.14	0	2 (1.7)	2 (1)
3.10	2 (2.5)	8 (6.7)	10 (5)
4.1	2 (2.5)	6 (5)	8 (4)
4.2	2 (2.5)	8 (6.7)	10 (5)
4.3	13 (16.2)	18 (15)	31 (15.5)
4.4	0	2 (1.7)	2 (1)
4.8	0	2 (1.7)	2 (1)
4.9	0	2 (1.7)	2 (1)
5.1	3 (3.8)	2 (1.7)	5 (2.5)
5.2	3 (3.8)	12 (10)	15 (7.5)
5.3	45 (56.2)	36 (30)	81 (40.5)
5.4	10 (12.5)	16 (13.3)	26 (13)
5.7	0	6(5)	6(3)
Total	80	120	200

Chi-Square = 24.991, df = 12, *p* = 0.015.

**Table 3:** Gender distribution of sagittal and vertical skeletal relationships.

Sagittal Skeletal Relationship *	**Gender ^a^**		**Total**
	Male	Female	
Class I	5 (6.2)	19 (15.8)	24 (12)
Class II	10 (12.5)	28 (23.3)	38 (19)
Class III	65 (81.2)	73 (60.8)	138 (69)
Total	80	120	200
Vertical Skeletal Relationship **	**Gender ^b^**		**Total**
	Male	Female	
High-angle	45 (56.2)	83 (69.2)	128 (64)
Normal	11 (13.8)	11 (9.2)	22 (11)
Low-angle	24 (30)	26 (21.7)	50 (25)
Total	80	120	200

* Assessed based of ANB angles: Class I (1 ≤ ANB ≤ 4), Class II (ANB > 4), and Class III (ANB < 1). ** Assessed based on GoGnSN angles: normal (28 ≤ GoGnSN ≤ 36), high-angle (GoGnSN> 36), and low angle (GoGnSN < 28). Chi-Square ^a^ = 9.538, df = 2, *p* = 0.008; Chi-Square ^b^ = 3.501, df = 2, *p* = 0.174.

**Table 4:** Gender distribution of IOFTN grades.

IOFTN Grades	Gender *		Total
	Male	Female	
≤3	2 (2.5)	10 (8.3)	12 (6)
4	17 (21.2)	38 (31.7)	55 (27.5)
5	61 (76.2)	72 (60)	133 (66.5)
Total	80	120	200

* Chi-Square = 6.522, df = 2, *p* = 0.038.

**Table 5:** Distribution of IOFTN grades according to sagittal and vertical skeletal relationships.

**IOFTN Grades**	Sagittal Skeletal Relationship *			**Total**
	**Class I**	**Class II**	**Class III**	
≤3	3 (12.5)	7 (18.4)	2 (1.4)	12 (6)
4	8 (33.3)	10 (26.3)	37 (26.8)	55 (27.5)
5	13 (54.2)	21 (55.3)	99 (71.7)	133 (66.5)
Total	30	32	138	200
**IOFTN Grades**	Vertical Skeletal Relationship **			**Total**
	**High-angle**	**Normal**	**Low-angle**	
≤3	8 (6.2)	2 (9.1)	2 (4)	12 (6)
4	31 (24.2)	8 (36.4)	16 (32)	55 (27.5)
5	89 (69.5)	12 (54.5)	32 (64)	133 (66.5)
Total	128	22	50	200

* Chi-Square = 18.405, df = 4, *p* = 0.001; ** Chi-Square = 2.891, df = 4, *p* = 0.576.

**Table 6:** Distribution of the osteotomy types in the sample.

Valid	Frequency (%)
BSSO *	12 (6)
BSSO + Genioplasty	12 (6)
Genioplasty	2 (1)
LeFort I	64 (32)
LeFort I + BSSO	72 (36)
LeFort I + BSSO+ Genioplasty	22 (11)
LeFort I + Genioplasty	16 (8)
Total	200

* BSSO: bilateral sagittal split osteotomy.
